# Treatment of Bright’s Disease

**Published:** 1887-03

**Authors:** 


### TREATMENT OF BRIGHT'S DISEASE.

Semmola, of Naples, in an article in the *Wiener Medizinische Blätter*, No 49, advises strongly against allowing a patient who is suffering from nephritis to come in contact with cold in any avoidable way. Such patients are extremely sensitive to cold, and cold baths are followed by great shock and depression. Violent massage and exercise of the muscles, the author strongly deprecates as followed by great shock and weakness.

He would advise the patient to live in a dry and equable climate; to strictly avoid all exposure, or going about in severe winter weather; to practice mild gymnastics in a comfortable room, rather than venture into a temperature below 18° or 20° C. The author emphasizes the remarkable sensibility of the skin of the sufferer with Bright's disease to all variations of temperature. Sodium iodide and chloride is advised in doses as large as tolerated. When, after two or three weeks, albumen has not entirely disappeared and dropsy has been relieved, phosphates of sodium or calcium are given in quantities as large as 40 grains or a drachm daily. The efficacy of these drugs the author believes consists in their power to promote the assimilation of albumen.

The methodical inhalation of oxygen, which Semmola has urged since 1867, has been repeatedly proved to be of the highest benefit. Albumen soon disappears after its use, and although casts may remain in the urine, the patient's general condition is so much improved that the author thinks we have here an argument for the dyscrasic or hæmatogenic origin of Bright's disease.

All astringents are considered not only valueless but also injurious. Especially is the action of ferrum sesquichloratum and plumbum aceticum thought injurious, because of their astringent influence on the capillaries of the skin.

